# Supplementary material for: Adoption of Electricity in Rural Rwanda 10 Years after Connection
Source: Nat Commun. 2025 Dec 7;16:10942. doi: 10.1038/s41467-025-66986-0 (PMC12686535; doi:10.1038/s41467-025-66986-0)
Supplement: Supplementary file 1 — Supplementary Information [file 41467_2025_66986_MOESM1_ESM.pdf]

Supplementary Information

for

Adoption of Electricity in Rural Rwanda 10 Years after Connection

Lise Masselus [1,2], Jörg Ankel-Peters[1,2], Gabriel Gonzalez Sutil[3], Vijay Modi[3], Joel Mugyenyi[3], Anicet Munyehirwe[4], Nathan Williams[5] & Maximiliane Sievert[1]*

*[1]RWI – Leibniz Institute for Economic Research, 45128 Essen, Germany. [2]University of Passau, 94030 Passau, Germany. [3]Columbia University, New York, NY 10027, United States of America. [4]IB&C, Kigali, Rwanda. [5]Rochester Institute of Technology, Rochester, NY 14623, United States of America. * email: lise.masselus@rwi-essen.de*

Contents

Supplementary Notes....3

Supplementary Note 1: Rural electrification in Rwanda 3

Supplementary Note 2: Definition of a community 4

Supplementary Note 3: the Next Generation Community-Based Environmental Assessment 5

Supplementary Figures 6

Supplementary References 7

Supplementary Notes

Supplementary Note 1: Rural electrification in Rwanda

The cornerstone of Rwanda's electrification endeavor is the Electricity Access Roll-out Program (EARP), nowadays run by the national utility Rwanda Energy Group (REG, previously the Energy, Water and Sanitation Authority or EWSA), which extends the grid through rural areas. In addition to supporting direct access, parts of the EARP funds were invested into extending transmission (or high-voltage) lines and improving grid stability. This paper focusses on the grid extension and grid densification activities carried out by EARP. Grid extension activities expand access to hitherto uncovered communities far from the existing grid by building new medium-voltage lines to communities, where power is transformed to low-voltage distribution lines. Grid densification activities connect communities and households closer to the grid by installing transformers from existing medium-voltage lines and extending low-voltage lines.

Once the grid reaches a community, households, enterprises, and social infrastructure can request a connection with REG. Connection fees are subsidized and determined by the distance to the grid. Currently, distances below 37 meters from the low-voltage line pay lower fees. According to conversations with REG in 2011, the distance where households can connect without additional expenses for extending distribution lines was 50 meters. For consistency of the sampling approach, we employ the 50 meters distance to determine the connection corridor. For households living close to the low-voltage line, the connection fee is 56,000 Rwandan Francs (RWF) (93 United States Dollar (USD)), which is roughly equivalent to 1.5 times the median household's monthly expenditures. Following Lee et al.[1], we call these "under-grid households" throughout the paper. For households living outside this corridor, connection fees increase as a function of distance to the existing low-voltage line, to cover the increasing cost of extending the distribution lines. For all households, connection fees can be paid in installments, which are added to each electricity bill. Since 2017, upfront payments are abolished for the poorest households. Most households receive a so-called ready board, a connection point ready for household use with two sockets and two light bulbs.

Households use a pre-paid meter to purchase electricity. These meters are recharged with tokens, unique numbers that can be purchased using mobile money or through a

commissioned REG agent in a local shop. Most households in rural areas recharge their prepaid meters frequently, on an as-needed basis and for small amounts.

Electricity tariffs per kilowatt-hour (kWh) have changed over the years. Between 2006 and 2015, tariffs for residential consumers increased from 112 RWF per kWh to 182 RWF per kWh to cover the cost of service and increased generation costs. Since 2017, tariffs for the lowest consumers have decreased by half to increase affordability for the poor. A block tariff is charged, where the first 15 kWh each month costs 89 RWF per kWh, the next 35 kWh costs 182 RWF per kWh and any kWh above 50 is charged at the highest prices of 210 RWF per kWh[2].

In light of the large number of new connections, capacity bottlenecks can be a major constraint that could affect the decision to connect and consume electricity[3,4]. In our sample, grid-connected households report to be satisfied with their connection. 79% of all households rate the quality of supply as good or excellent and only 1% rate the quality of supply as poor.

Blackouts and voltage fluctuations occur, but relatively infrequently compared to other SSA countries[3]. In the month prior to our 2022 survey, 61% of connected households report to have experienced blackouts, which occur once per week on average. Blackouts last 3.6 hours on average. 21% of all connected households reported that they had noticed voltage fluctuations, which occur only once per month or less in 70% of the cases. Blimpo & Cosgrove-Davies[3] document for eleven countries, including neighboring countries Burundi, Tanzania, and Uganda, that over 40% of the connected households experience outages for over half of the time. In Kenya, approximately one fourth of connected households have outages for over half of the time. Bensch et al.[5] study the service quality in two grid-covered towns in rural Tanzania. Blackouts are reported by 88% of all connected households, occur twice per week and last two to eight hours on average.

Supplementary Note 2: Definition of a community

We define a community as a group of households clustered around basic infrastructure. We resort to this definition of a community, since standardized administrative units in Rwanda are not helpful for thinking about electrification projects. Low-voltage lines are most commonly constructed in small centers. These centers are often located at the intersection of several imidugudu, the lowest administrative unit. Typically, one side of the road belongs to one umudugudu and the opposite side to a different one. Sometimes, three or four imidugudu

intersect in the center. The next higher administrative level (*cell*) bundles around seven imidugudu on average and is therefore too coarse. This is why we chose to cluster several imidugudu together into what we call "communities", based on the situation on the ground. For all households within such a community, the center used for community definition is usually the nearest access point to the electricity grid.

Supplementary Note 3: the Next Generation Community-Based Environmental Assessment

The "electricity as a right" perspective aligns with the principles of the Next Generation Community-Based Environmental Assessment (NG-CBEA), which emphasizes the importance of comprehensive assessments, meaningful participation of beneficiaries, and the consideration of normative principles in evaluating development interventions[6]. This also means embracing logical and practical interdependencies between effectiveness, efficiency, and fairness.

Supplementary Figures

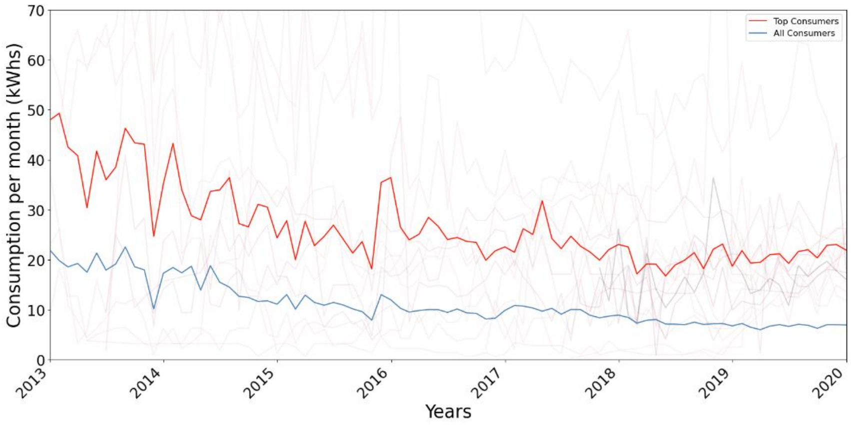

Supplementary Fig. 1: **Electricity consumption over time (kWh) for matched households**. Source: Administrative consumption data. N = 174 for all consumers. “Top consumers” indicate the highest 10% of consumers (N=17) in our matched sample.

Supplementary References

1. Lee, K. *et al.* Electrification for "under grid" households in rural Kenya. *Dev. Eng.* **1**, 26–35 (2016).

2. Mugyenyi, J. *et al.* Rwanda's path to universal electricity access: consumption trends, tariff impact, and challenges ahead. *SSRN Electron. J.* (2024).

3. Blimpo, M. P. & Cosgrove-Davies, M. *Electricity Access in Sub-Saharan Africa: Uptake, Reliability, and Complementary Factors for Economic Impact*. (World Bank Publications, Washington DC, 2019).

4. Meeks, R. C., Omuraliev, A., Isaev, R. & Wang, Z. Impacts of electricity quality improvements: experimental evidence on infrastructure investments. *J. Environ. Econ. Manag.* **120**, 102838 (2023).

5. Bensch, G. *et al. Electrifying Rural Tanzania. A Grid Extension and Reliability Improvement Intervention*. (2019).

6. Biswal, R., Sinclair, A. J. & Spaling, H. Moving to next generation community-based environmental assessment. *Impact Assess. Proj. Apprais.* **41**, 416–427 (2023).
