# Supplementary material for: Adoption of Electricity in Rural Rwanda 10 Years after Connection
Source: Nat Commun. 2025 Dec 7;16:10942. doi: 10.1038/s41467-025-66986-0 (PMC12686535; doi:10.1038/s41467-025-66986-0)
Supplement: Supplementary file 2 — Reporting Summary [file 41467_2025_66986_MOESM2_ESM.pdf]

nature portfolio

Corresponding author(s): Masselus, Lise

Last updated by author(s): Oct 28, 2025

Reporting Summary

Nature Portfolio wishes to improve the reproducibility of the work that we publish. This form provides structure for consistency and transparency in reporting. For further information on Nature Portfolio policies, see our Editorial Policies and the Editorial Policy Checklist.

Statistics

For all statistical analyses, confirm that the following items are present in the figure legend, table legend, main text, or Methods section.

n/a	Confirmed	
☐	☒	The exact sample size (*n*) for each experimental group/condition, given as a discrete number and unit of measurement
☐	☒	A statement on whether measurements were taken from distinct samples or whether the same sample was measured repeatedly
☒	☐	The statistical test(s) used AND whether they are one- or two-sided<br>*Only common tests should be described solely by name; describe more complex techniques in the Methods section.*
☐	☒	A description of all covariates tested
☐	☒	A description of any assumptions or corrections, such as tests of normality and adjustment for multiple comparisons
☐	☒	A full description of the statistical parameters including central tendency (e.g. means) or other basic estimates (e.g. regression coefficient) AND variation (e.g. standard deviation) or associated estimates of uncertainty (e.g. confidence intervals)
☒	☐	For null hypothesis testing, the test statistic (e.g. *F*, *t*, *r*) with confidence intervals, effect sizes, degrees of freedom and *P* value noted<br>*Give P values as exact values whenever suitable.*
☒	☐	For Bayesian analysis, information on the choice of priors and Markov chain Monte Carlo settings
☒	☐	For hierarchical and complex designs, identification of the appropriate level for tests and full reporting of outcomes
☐	☒	Estimates of effect sizes (e.g. Cohen's *d*, Pearson's *r*), indicating how they were calculated

Our web collection on statistics for biologists contains articles on many of the points above.

Software and code

Policy information about availability of computer code

Data collection	survey CTO
Data analysis	STATA 15.1, R 1.4.1103, Python 3.11.0, Microsoft Excel

For manuscripts utilizing custom algorithms or software that are central to the research but not yet described in published literature, software must be made available to editors and reviewers. We strongly encourage code deposition in a community repository (e.g. GitHub). See the Nature Portfolio guidelines for submitting code & software for further information.

Data

Policy information about availability of data

All manuscripts must include a data availability statement. This statement should provide the following information, where applicable:

- Accession codes, unique identifiers, or web links for publicly available datasets
- A description of any restrictions on data availability
- For clinical datasets or third party data, please ensure that the statement adheres to our policy

All survey data and code is stored on the OSF platform (https://doi.org/10.17605/OSF.IO/XQYST). The administrative data cannot be shared due to privacy issues. Access might be granted after applying at info@reg.rw.
Data used from the Multi-Tier Framework (MTF) Surveys is provided by the World Bank Group and accessed via https://energydata.info/dataset. The core datasets for Rwanda, Kenya, Nigeria, Zambia, and Ethiopia were downloaded on September 6, 2021. The dataset for Niger was downloaded on March 29, 2021, and the

dataset for Liberia was downloaded on September 20, 2021. Additional variables—specifically household weights and unique identifiers—for Ethiopia, Liberia, and Nigeria were not available at the time of original download and were subsequently retrieved on August 15, 2022. The datasets are available under the Creative Commons Attribution 4.0 and the CC0 1.0 license.

Research involving human participants, their data, or biological material

Policy information about studies with human participants or human data. See also policy information about sex, gender (identity/presentation), and sexual orientation and race, ethnicity and racism.

Reporting on sex and gender	The analysis looks at households and villages and does not perform any analyses for individual household members, We therefore do not study any questions related to sex or gender.
Reporting on race, ethnicity, or other socially relevant groupings	We do not perform any analyses regarding race, ethnicity, or other socially relevant groupings.
Population characteristics	Study communities are located across rural Rwanda. We define a community as a group of households, clustered around basic infrastructure. One community might cover mutliple administrative settlements, so-called umudugudus. In 2011 the average population is 300 households per community. All communities are in rural areas, where the majority relies on farming as their primary income sources. Community-level data indicate limited prior enterprise activity and low infrastructure access. Detailed demographic characteristics such as age, gender, and education of household heads were collected to serve as covariates in analyses. (See “Behavioural & social sciences study design” for further detail.)
Recruitment	Participants were recruited through in-person visits to communities and systematic household selection along distribution lines. Recruitment relied on voluntary participation with informed consent. Potential biases include self-selection bias, as households willing to participate may differ slightly in socio-economic status or grid connection likelihood. These biases are minor and unlikely to substantially affect aggregate results, given that hardly any household refused participation.
Ethics oversight	University of Connecticut IRB (U Conn IRB #20-0104)

Note that full information on the approval of the study protocol must also be provided in the manuscript.

Field-specific reporting

Please select the one below that is the best fit for your research. If you are not sure, read the appropriate sections before making your selection.

☐ Life sciences ☒ Behavioural & social sciences ☐ Ecological, evolutionary & environmental sciences

For a reference copy of the document with all sections, see nature.com/documents/nr-reporting-summary-flat.pdf

Behavioural & social sciences study design

All studies must disclose on these points even when the disclosure is negative.

Study description	The study employs a quantitative research design that analyzes long-term electricity adoption patterns in rural Rwanda. It combines self-collected household and community survey data from four waves (2011, 2013, 2015, and 2022) with administrative electricity consumption data from the Rwanda Energy Group (REG) and World Bank Multi-Tier Framework (MTF) datasets. The triangulation of these quantitative sources allows for a robust examination of connection rates, electricity consumption, and appliance use over time, providing evidence on adoption dynamics a decade after grid connection.
Research sample	The study uses three data sources. First, it includes around 800 households across 41 rural Rwandan communities electrified under the first phase of the Electricity Access Roll-out Program (EARP). The communities are selected to be representative of rural areas scheduled for electrification between 2009 and 2013. The average community comprises about 300 households, most of whom rely on farming as their main source of income. The average household comprises five members, typically headed by a male in his forties with primary or secondary education. Women-headed households make up a notable share of the sample, reflecting rural Rwanda’s demographic structure. The sample represents “under-grid” households—those located within 50 meters of distribution lines. The sample is chosen to capture long-term effects of grid coverage on communities with direct access to electricity infrastructure. Second, in addition to the survey data, the study uses administrative electricity consumption data from the Rwanda Energy Group (REG), which include purchase records for approximately 800,000 consumers, of which 147,074 rural households are identified and retained for analysis. This dataset provides a comprehensive view of actual electricity usage between October 2012 and April 2020 and allows validation of survey-based consumption estimates. Third, the study incorporates data from the World Bank’s Multi-Tier Framework (MTF) surveys, collected between 2016 and 2018 for six Sub-Saharan African countries—Rwanda, Ethiopia, Niger, Nigeria, Kenya, and Zambia. These nationally representative datasets cover rural grid-connected households and provide comparative information on appliance ownership and electricity use, enabling assessment of the generalizability of the Rwandan findings within the regional context.
Sampling strategy	The communities are selected to be representative of rural areas scheduled for electrification between 2009 and 2013. Households are selected using a random walk sampling procedure designed to yield a representative cross-section of all under-grid households—those located within 50 meters of the low-voltage distribution lines. Upon arrival in a community, survey teams identify the eligible corridor, estimate the total number of households, and interview every xth household based on the ratio of total

households to the intended sample size (30 in 2011, 20 in 2022). This procedure ensures random selection and avoids interviewer bias. No formal statistical power calculation is performed prior to data collection. Instead, sample sizes are chosen to achieve community-level representativeness and allow for meaningful subgroup analysis within the limits of available resources. The chosen sample size of 820 households across 41 communities is sufficient to capture reliable variation in connection and consumption behavior while maintaining logistical feasibility.

Data collection

Data were collected through structured household and community surveys conducted by trained field teams in collaboration with IB&C Rwanda in 2011, 2013, 2015 and 2022. A random walk sampling method was used to identify households under the grid corridor. Additional data comes from administrative consumption records (2012–2020) provided by REG, as well as from MTF datasets used for cross-country comparisons. The instruments used included pre-tested questionnaires capturing information on electricity usage, appliance ownership and socio-economic indicators. Until 2015, all interviews were performed using pen and paper. The 2022 interviews were conducted using CAPIs. Only researchers and enumerators are present during interviews. As this is an observational, non-experimental study, researcher blinding is not

Timing

Data collection occurred in four main waves: 2011, 2013, 2015, and 2022. The administrative consumption data covered October 2012 to April 2020. The earliest surveyed communities were electrified around 2009–2013, allowing for up to ten years of follow-up data. There were gaps between survey waves corresponding to the periods between 2015 and 2022, when no household survey data were collected.

Data exclusions

Two non-electrified communities from the original Lenz et al. (2017) sample were excluded, as they were not covered by the grid and thus not relevant for this analysis. No household-level data were excluded.

Non-participation

Refusal in survey was negligible

Randomization

The study does not include experimental groups, as it is designed as a non-experimental, observational follow-up study assessing long-term adoption of electricity after a nationwide grid expansion program. Consequently, no random allocation or control group assignment takes place.

Reporting for specific materials, systems and methods

We require information from authors about some types of materials, experimental systems and methods used in many studies. Here, indicate whether each material, system or method listed is relevant to your study. If you are not sure if a list item applies to your research, read the appropriate section before selecting a response.

Materials & experimental systems

n/a	Involved in the study
☒	☐ Antibodies
☒	☐ Eukaryotic cell lines
☒	☐ Palaeontology and archaeology
☒	☐ Animals and other organisms
☒	☐ Clinical data
☒	☐ Dual use research of concern
☒	☐ Plants

Methods

n/a	Involved in the study
☒	☐ ChIP-seq
☒	☐ Flow cytometry
☒	☐ MRI-based neuroimaging

Plants

Seed stocks

Report on the source of all seed stocks or other plant material used. If applicable, state the seed stock centre and catalogue number. If plant specimens were collected from the field, describe the collection location, date and sampling procedures.

Novel plant genotypes

Describe the methods by which all novel plant genotypes were produced. This includes those generated by transgenic approaches, gene editing, chemical/radiation-based mutagenesis and hybridization. For transgenic lines, describe the transformation method, the number of independent lines analyzed and the generation upon which experiments were performed. For gene-edited lines, describe the editor used, the endogenous sequence targeted for editing, the targeting guide RNA sequence (if applicable) and how the editor was applied.

Authentication

Describe any authentication procedures for each seed stock used or novel genotype generated. Describe any experiments used to assess the effect of a mutation and, where applicable, how potential secondary effects (e.g. second site T-DNA insertions, mosiacism, off-target gene editing) were examined.
